# Increased extracellular water/body mass is associated with functional impairment in hemodialysis patients

**DOI:** 10.1080/0886022X.2023.2271066

**Published:** 2023-11-21

**Authors:** Lin Zhan, Lu Liu, Jing Yuan, Chaomin Zhou, Yan Zha

**Affiliations:** a Central laboratory of Guizhou Provincial People’s Hospital, Guiyang, China; b NHC Key Laboratory of Pulmonary Immune-related Diseases, Renal Division, Department of Medicine, Guizhou Provincial People’s Hospital, Guiyang, China; c Guizhou University Medical College, Guiyang, China

**Keywords:** Extracellular water and body cell mass ratio, the Karnofsky performance status score, hemodialysis, fluid overload

## Abstract

**Background:**

Functional impairment, malnutrition and fluid overload are prevalent in patients undergoing maintenance hemodialysis (MHD). The extracellular water/body cell mass ratio (ECW/BCM) is a new indicator reflecting fluid overload and malnutrition. A previous study has suggested that it performs better than other indices in assessing fluid status. This study investigates the relationship between pre-dialysis whole-body ECW/BCM and physical function in MHD patients.

**Methods:**

We conducted a multicenter, cross-sectional study in Guizhou Province in Southwest China. The Karnofsky Performance Status (KPS) was used to evaluate patients’ functional status. Patients with KPS scores of ≤ 80 were considered to have a functional impairment. The body composition was measured using the body composition monitor (BCM), and the value of the ECW/BCM ratio was calculated. The subjects were classified into three groups according to ECW/BCM tertiles. Multiple logistic regression models and interactive analyses were conducted.

**Results:**

The final analysis included 2818 subjects. Multivariate logistic regression analyses showed that compared with the lowest tertile (tertile 1), the adjusted odds ratio of functional impairment were 1.95 (95% CI: 1.21–3.13, *p* < 0.001) and 2.10 (95% CI: 1.31–3.37, *p* < 0.001) in the second and the third tertiles of ECW/BCM, respectively after adjusting for age, sex, current smoking status, history of stroke, heart failure, diabetes, and myocardial infarction. Subgroup analysis showed that the association existed stably across all subgroups stratified by age, gender, cognitive impairment (CI), history of stroke, heart failure, and diabetes (all p values for interaction >0.05).

**Conclusions:**

Elevated ECW/BCM is independently linked to an increased risk of functional impairment in patients with MHD.

## Introduction

Functional impairment is prevalent in patients receiving maintenance hemodialysis (MHD) treatment [1]. Impaired physical function is associated with reduced quality of life [2] and an increased risk of all-cause morbidity and mortality in patients on dialysis [3]. Therefore, identifying modifiable factors may aid in developing preventive strategies to reduce the morbidity and mortality associated with functional impairment in this population.

Fluid overload (FO) and malnutrition are common complications in dialysis patients. They not only lead to unpleasant symptomatology for dialysis patients but also result in various adverse outcomes such as heart failure, pulmonary edema, hospitalization, and even death [4]. Therefore, managing volume balance and nutritional status is essential in this population. Bioimpedance spectroscopy (BIS)-derived extracellular water/body cell mass ratio (ECW/BCM) has been proposed as an indicator that reflects fluid overload and malnutrition simultaneously in patients undergoing renal replacement therapy [5]. Previous studies have suggested the ECW/BCM as a potential predictive factor for mortality risk in HD patients [6]. The ECW/BCM was found to perform better than overhydration and extracellular water (OH/ECW) in the assessment of fluid status in patients with acute kidney injury who required renal replacement therapy [7] as well as in those undergoing maintenance hemodialysis (MHD) [8]. Unfortunately, little is known about the relationship between the whole-body ECW/BCM ratio before dialysis and functional capabilities in MHD patients. We hypothesized that an increased pre-dialysis whole-body ECW/BCM ratio may be associated with functional impairment in MHD patients.

The Karnofsky Performance Status (KPS) has been widely used to assess the functional status of patients with various conditions, including chronic kidney disease [9]. Therefore, this study investigated the relationship between pre-dialysis whole body ECW/BCM ratio and physical function assessed with KPS scores in MHD patients.

## Materials and methods

### Study design and participants

We conducted a multicenter, cross-sectional study from 1 June 2020 to 30 September 2020 in Guizhou Province, Southwest China. All adult HD patients in 20 tertiary hospital dialysis centers of Guizhou Province who have been on regular hemodialysis for at least three months were invited to participate in our study. Subjects were excluded from the study if they met one or more of the following criteria: (1) they were fitted with a cardiac pacemaker; (2) with limb defects; (3) younger than 18 years of age; (4) with a language barrier; (5) mentally handicapped; (6) visually or auditorily impaired; (6)patients with no routine test results in the last three months; (7) patients on diuretic and corticosteroid medications, which may interfere with nutritional hydration status; (8) patients had a severe medical condition, such as those with ongoing cardiac failure, gastrointestinal bleeding. The study was approved by the ethics committee of Guizhou Provincial People’s Hospital for ethical approval (approval number: GZPPHEC-2015-028). All participants provided written informed consent for participation before study enrollment.

### Data collection

As described in our previous studies, a standardized structured interview was used to collect demographic information, medical history, and lifestyle details [10,11]. All MHD subjects were required to have a routine blood draw performed at least once every three months, which included a biochemical test and routine blood work. Laboratory data such as serum uric acid (SUA), serum creatinine (Scr), and albumin levels were abstracted from routine blood tests and were used in the analysis. Only the most up-to-date data were analyzed.

### Anthropometric measurement and body composition analysis

Following a routine HD session, anthropometric measurements of height, weight, and waist circumference were carried out by well-trained staff. During anthropometric measurements, all participants were instructed to stand barefoot and wear light clothing. Body mass index (BMI) was calculated as the body weight (kg) divided by the square of the body height (m). All body composition analyses were performed by trained staff with a portable body composition monitor (BCM, Fresenius Medical Care, Bad Homburg, Germany). BCM, ECW, and ICW were evaluated by the multifrequency bioimpedance method using the BCM. Body composition analysis was performed approximately 30 min before dialysis treatment with the patient in a supine position.

### Functional status assessment

The KPS scale was used instead of the performance status of the Eastern Cooperative Oncology Group (ECOG) to evaluate patients’ functional status since the KPS is more sensitive in identifying functional status. In addition, ECOG is validated explicitly in the cancer population [12]. Based on previous studies, patients with a KPS score of ≤ 80 were considered to have functional impairment [13]. Patients’ cognitive function was also assessed in our study using the mini-mental state examination (MMSE) score. Patients with MMSE scores less than 27 were considered to have cognitive impairment (CI) [14].

### Statistical analysis

The subjects were divided into tertiles according to the ECW/BCM ratio, and the patients’ characteristics were compared between the groups. For continuous variables, data were reported as the mean ± standard deviation when normally distributed or as the median (IQR) when the distribution was asymmetric. Data were presented as percentages for categorical variables. Comparisons of differences between tertiles were made using One-way ANOVA or the Kruskal-Wallis test for continuous variables, while chi-square tests were used to analyze categorical variables. Univariable logistic regression analyses were used to identify parameters associated with impaired functional status. Then, to determine the independent association of the ECW/BCM ratio with functional impairment, multivariable logistic regression analyses were performed with covariates *p* < 0.05 from the univariate logistic regression analyses. Subgroup analyses were conducted to assess the robustness of the primary findings. In subgroup analyses, we used the likelihood ratio test to test whether there was interaction. All data analysis was done with the software package SPSS 20.0 (SPSS Inc., Chicago, IL, USA). A P value <0.05 is considered to indicate statistical significance.

## Results

### Baseline characteristics

Our initial study included 3312 subjects. Data from 494 subjects were excluded from the final analysis because of missing data for ECW, BCM, and KPS scores. We ultimately included 2818 subjects in our final analysis. The mean age of the patients was 54 ± 15 years. Table 1 shows the baseline characteristics of the study participants stratified by ECW/BCM tertiles. Patients in the higher ECW/BCM ratio quartiles tended to be older and had lower levels of Scr, SUA, albumin, BCM, handgrip strength and higher levels of BMI and ECW. They also had a higher prevalence of diabetes, hypertension, stroke, and heart failure history. The prevalence of functional impairment increased with higher ECW/BCM tertiles (3.5%, 9.5%, and 17.5% in the first, second, and third tertile, respectively; *p* < 0.05).

**Table 1.** Clinical characteristics of the participants stratified by ECW/BCM tertiles.

Variables	Tertile 1 (*n* = 926)	Tertile 2 (*n* = 958)	Tertile 3 (*n* = 934)	P
Age (years)	48 ± 14	55 ± 14	61 ± 14	< 0.001
Male (%)	630 (68)	536 (55.9)	483 (51.7)	< 0.001
Current tobacco use (%)	266 (29)	211 (22.1)	184 (20)	< 0.001
Current drinker (%)	55 (6)	48 (5)	44 (4.8)	0.46
History of hypertension (%)	679 (75.3)	746 (78.9)	730 (79.4)	0.065
History of diabetes (%)	123 (14.7)	254 (28.7)	413 (47.6)	< 0.001
History of stroke (%)	48 (5.3)	74 (8)	130 (14.2)	< 0.001
History of heart failure (%)	191 (21.2)	260 (28)	301 (33.1)	< 0.001
CI(%)	130 (14.1)	220 (23.2)	293 (31.5)	< 0.001
Handgrip strength (kg)	24.9 ± 10.3	21.0 ± 9.3	18.2 ± 9.1	< 0.001
Systolic blood pressure (mm Hg)	138 (125,151)	138(124, 151)	139(124, 152)	0.544
Diastolic blood pressure (mm Hg)	82 (74, 91)	78 (70, 87)	75 (66, 85)	< 0.001
BMI (kg/m^2^)	22.2 ± 3.4	22.8 ± 3.4	23.3 ± 4.2	< 0.001
LTM(kg)	46.0 ± 9.4	39.1 ± 7.5	32.2 ± 7.1	< 0.001
CRP (mg/L)	2.0 (0.9, 4.1)	2.2 (1.1, 6.2)	3.1 (1.6, 7.0)	< 0.001
ECW/BCM (L/kg)	0.5 ± 0.1	0.7 ± 0.0	0.9 ± 0.3	<0.001
ICW (L)	20.5 ± 3.7	18.1 ± 3.3	15.9 ± 3.1	<0.001
ECW(L)	14.5 ± 2.7	14.6 ± 2.9	15.1 ± 4.5	<0.001
Functional impairment (%)	30 (3.5)	83 (9.5)	152 (17.5)	< 0.001
Serum creatinine (µmol/L)	1068.0 (882.0, 1250.0)	930.0 (747.3, 1114.0)	802.3 (631.8, 977.4)	< 0.001
Serum uric acid (µmol/L)	455.3 (389.0, 528.9)	444.0 (373.3, 519.5)	421.0 (356.6, 489.0)	< 0.001
Albumin (g/L)	41.1 (38.7, 43.3)	40.2 (38.0, 42.7)	39.5 (36.8, 42.3)	< 0.001

ECW: extracellular water; ICW: intracellular water; BMI: body mass index; CRP: C-reaction protein; CI: cognitive impairment; LTM: lean tissue mass.

### Association between increased ECW/BCM ratio and functional impairment

As shown in Table 2, the univariate logistic regression models indicated that ECW/BCM, age, gender, CI, history of diabetes, stroke, heart failure, and diabetes were significantly associated with a greater risk of functional impairment (*p* < 0.05 for all). As shown in Table 3, the unadjusted OR of functional impairment was 2.89 (95% CI:1.88-4.44) and 5.84 (95% CI: 3.90–8.75) for those in tertile 2 and tertile 3, respectively, compared with the patients in tertile 1. Multiple logistic regression analyses were conducted to determine the independent impact of increased ECW/BCM ratio on functional impairment. Despite our data not yielding statistically significant associations between continued smoking and dialysis vintage with functional impairment, detrimental effects of smoking status and dialysis vintage on cardio-respiratory fitness need to be considered. We, therefore, adjusted for both of these potential confounders in our subsequent analyses. After adjusting for age, sex, current smoking status, history of stroke, heart failure, diabetes, and myocardial infarction, patients in tertile 2 were found to have a fold increase of 1.95 (95% CI: 1.21 to 3.13, *p* < 0.001) and a 2.10 fold increase (95% CI: 1.31-3.37, *p* < 0.001) in tertile 3 as likely to have functional impairment compared to those in tertile 1. These associations persisted even after adjustment for CI and dialysis vintage.

**Table 2.** The potential risk factors for functional impairment by univariable logistical regression.

Variables	OR	95% CI	P value
ECW/BCM	3.01	1.91–4.74	0.001
Female gender	1.35	1.05–1.74	0.013
Age	1.07	1.06–1.08	<0.001
Educational status (≥high school) (%)	0.95	0.71–1.26	0.183
History of hypertension	1.24	0.89 ∼ 1.71	0.453
Ongoing tobacco use	0.73	0.53–1.01	0.06
Current drinker	0.72	0.37–1.40	0.34
History of diabetes	2.68	2.06–3.49	<0.001
History of stroke	4.57	3.32–6.30	<0.001
History of heart failure	2.49	1.92 ∼ 3.24	<0.001
History of myocardial infarction	2.59	1.72–3.90	<0.001
Cognitive impairment	2.02	1.54–2.64	<0.001
Dialysis vintage	1	1–1	0.435
BMI	1.02	0.99–1.05	0.23
Handgrip strength	0.92	0.90–0.94	<0.001

**Table 3.** Association between increased ECW/BCM ratio and functional impairment.

Variable	Model 1	P value	Model 2	P value	Model 3	P value	Model 4	P value
Tertile 1	Reference		Reference		Reference		Reference	
Tertile 2	2.89 (1.88–4.44)	<0.001	2.13 (1.35–3.37)	0.002	1.95 (1.21–3.13)	0.007	1.89 (1.17–3.05)	0.009
Tertile 3	5.84 (3.9–8.75)	<0.001	2.98 (1.91–4.66)	<0.001	2.10 (1.31–3.37)	0.002	2.00 (1.24–3.22)	0.004

Model 1, unadjusted model

Model 2, adjusted for age and gender + ongoing tobacco use

Model 3, above + history of stroke, heart failure, diabetes and myocardial infarction

Model 4, above + cognitive impairment + dialysis vintage.

### Analysis of subgroups

**Figure 1.**
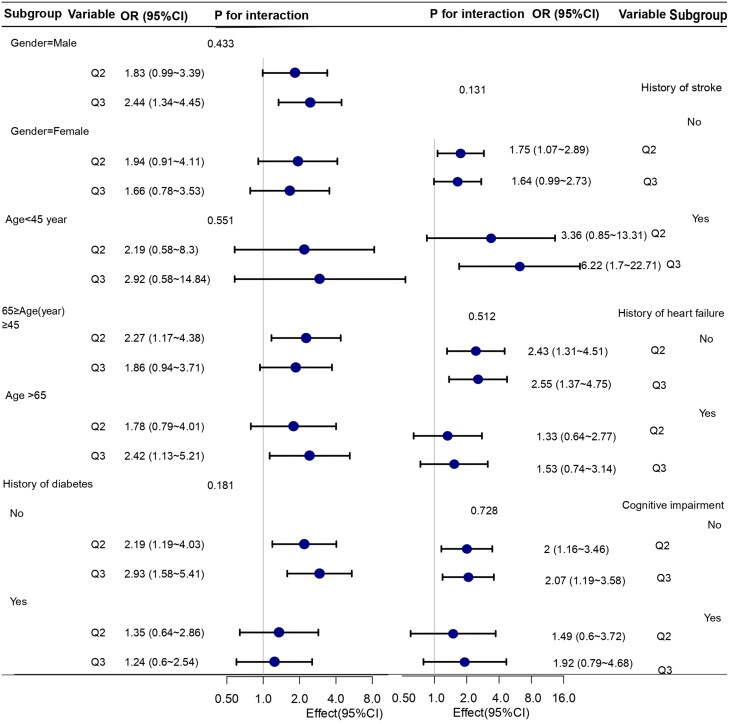
Subgroup analyses examining the association between ECW/BCM and functional impairment. Odds ratios (ORs) were calculated after adjusting for age, sex, history of diabetes, stroke, heart failure, myocardial infarction and cognitive impairment.

The influence of older age, poorer cognitive function, and female gender on subjects’ functional status is well recognized [15]. To assess the robustness of the primary outcomes, we performed subgroup analyses to assess potential effect modification by age, sex, CI, history of stroke, heart failure, and diabetes. For further analysis of the impact of age, we classified participants into three subgroups according to their age (< 45,≥45, and < 65; ≥65 years of age). As shown in Figure 1, the associations of ECW/BCM with functional impairment were similar across all subgroups (all p values for interaction >0.05).

## Discussion

The present study is the first to investigate the relationship between the pre-dialysis whole-body ECW/BCM ratio and functional capacity. In this multicenter cross-sectional study, we found that an increased ECW/BCM ratio was associated with a high risk of functional impairment in patients with MHD, independent of age, sex, current smoking status, dialysis vintage, and prior medical history. Similar results were found in subgroup analyses.

The BIS-derived indexes have received increasing attention in recent years. It has been reported that the ECW/BCM ratio is a sensitive marker for nutritional assessment in patients with chronic radiation enteritis and tumors [16,17]. In addition to being a novel marker of being wasted-overhydrated in the dialysis population [5], the ECW/BCM ratio is also an independent predictor of survival in patients with end-stage renal disease (ESRD) who have undergone either peritoneal dialysis (PD) or HD treatment [5]. Extracellular mass is mainly comprised of extracellular fluid. It has been suggested in previous studies that the ECW/BCM performed better than the OH/ECW as well as the ratio of ECW to intracellular water (ECW/ICW) in the assessment of fluid status [7,8]. Higher ECW/ICW ratios are associated with functional disability levels in patients with knee osteoarthritis [18]. HD patients are at particularly high risk for physical and functional impairment. No study has previously investigated the association between the ECW/BCM ratio and functional impairment in this particular population.

Our results indicate that higher ECW/BCM is associated with a greater risk of functional impairment as assessed by KPS in patients with MHD. In a previous study, fluid overload before dialysis was associated with slower and decreased gait speed over time in chronic dialysis patients [19], which is similar to our findings, as the increased ECW/BCM ratios reflect water overload. An ECW/BCM ratio increase may result from increased ECW volume or a reduction in BCM, composed of protein and ICW. This is supported by our findings that although participants in the highest ECW/BCM tertile group had the highest levels of BMI, these patients had the lowest levels of LTM, ICW, and highest levels of ECW. It has been suggested in a previous study that ECW/ICW in the upper legs is negatively associated with skeletal muscle strength and gait velocity, which reflects muscle strength and physical function in the elderly [20]. Another study has suggested increased whole-body ECW/ICW in community-dwelling older women is associated with decreased handgrip strength and gait speed [21]. These findings are similar to ours. Muscle strength determined by grip strength and muscle mass determined by LTM in our study decreased with increasing ECW/BCM tertiles. Although it is well known that physical activity and physical function can be highly variable between different sexes and decline with age, prior studies included only older and female subjects. No previous study investigated the association between fluid overload and functional impairment in different age and gender subgroups. In addition, comorbidities such as cognitive impairment, diabetes mellitus [22], a history of stroke, and heart failure may also contribute to impaired physical function, while previous studies did not adjust for these confounders. The potential heterogeneity between different subgroups should be taken into account. To explore the association between increased ECW/BCM and functional impairment in hemodialysis patients, we conducted subgroup analyses stratified by age, sex, CI, history of stroke, heart failure, and diabetes. We found similar results in different subgroups.

The mechanisms accounting for the increased risk of functional impairment with higher ECW/BCM in MHD patients have yet to be elucidated, but several plausible mechanisms could be speculated. Increased ECW/BCM is a marker that simultaneously reflects fluid overload and malnutrition. Participants in the highest tertile of the ECW/BCM group may have had intestinal edema or decreased appetite, which results in deficiencies of essential nutrients such as protein and vitamin D. Protein and vitamin D have been shown to play a critical role in the preservation of muscle mass and strength [23]. Our findings that Scr, SUA, albumin, grip strength, and LTM decreased with increasing tertiles of ECW/BCM further support this hypothesis. Physical activity and function may thus decrease due to muscle weakness. It is also possible that patients with increased ECW/BCM may have more comorbidities, such as pulmonary edema, heart failure, and lower extremity edema, all of which contribute to reduced physical activity and function. Our findings that patients in higher ECW/BCM groups were more likely to have comorbidities such as diabetes and a history of heart failure align with this assumption. Thus, participants may experience a functional impairment due to physical inactivity. In addition, chronic low-grade inflammation plays a significant role in muscle weakness and frailty, which can worsen functional impairment. Our findings that C-reactive protein (CRP) levels in the tertile 3 group were higher than those in the lower tertile ECW/BCM groups provide further support for this hypothesis. Malnutrition, inflammation, and fluid overload may interact, leading to a vicious cycle and more severe functional impairment.

The primary strength of the present study was its multicenter representation of a southwest province of China. The second major strength of our study was the relatively large sample size, allowing for subgroup analyses. However, several limitations of our study should be considered when interpreting our results. First, the cross-sectional nature of the current study disenabled us to infer causal relationships between an increase in ECW/BCM and functional impairment. Second, physical activity or exercise training plays a vital role in muscle health, which is critical for physical functioning. However, data on physical activity were unavailable and not adjusted for in our study. Additional studies that consider the effects of physical activity are required to more clearly elucidate the associations of increased ECW/BCM with functional impairment. Third, serial measurement of the ECM/BCM ratio concerning potential longitudinal changes in KPS is required. Fourth, we did not compare the association between ECW/BCM and functional impairment with other BIS-derived fluid overload markers such as OH/ECW. Additional studies are needed to compare which marker best identifies functional impairment risk in MHD patients.

In conclusion, elevated ECW/BCM was independently associated with an increased risk of functional impairment in patients with MHD. Clinicians need to monitor ECW/BCM in MHD patients closely. Maintaining an adequate nutritional status over time and volume balance may help reduce functional impairment in MHD patients.

## Data Availability

Data is available from the corresponding author upon reasonable request.
